# Mothers’ daily perceived stress influences their children’s mental health during SARS-CoV-2-pandemic—an online survey

**DOI:** 10.1186/s13034-021-00385-3

**Published:** 2021-06-16

**Authors:** Franziska Köhler-Dauner, Vera Clemens, Stephanie Lange, Ute Ziegenhain, Jörg M. Fegert

**Affiliations:** Department of Child and Adolescent Psychiatry/Psychotherapy, University Hospital of Ulm, University of Ulm, Steinhövelstraße 5, 89075 Ulm, Germany

**Keywords:** SARS-CoV-2-pandemic, Emotional and social development of children and adolescents, Mental stress, Life quality, Psychosocial impact

## Abstract

**Background:**

The current situation caused by the SARS-CoV-2-pandemic is associated with serious losses for everyone and has been affecting social life, politics, the economy and the media worldwide. Preventive isolation and social distancing strategies have confronted families with a large number of different challenges. The current epidemic and quarantine restrictions have a verifiable influence on the emotional and social development of children and adolescents. During this ongoing situation children of parents, who already were mentally stressed, seem particularly at risk.

**Objective:**

We aimed to assess the role of maternal daily perceived stress on children’s mental health during the SARS-CoV-2-pandemic.

**Methods:**

An online “SARS-CoV-2-pandemic survey” was developed to assess children’s mental health since the beginning of the SARS-CoV-2 pandemic. To describe maternal perceived everyday stress, data from a longitudinal survey was utilized. Our survey includes elements and versions of the Childhood Trauma Questionnaire, the Strengths and Difficulties Questionnaire and the Perceived Stress Scale. We furthermore collected socio-demographic data. Due to our limited dependent variables we applied Tobit models for estimation.

**Results:**

We found a positive and significant effect of the maternal perceived everyday stress on children’s emotional problems during the pandemic. Furthermore, results provide empirical evidence for an increase of the children's hyperactivity level dependent on the mother’s perceived stress before the SARS-COV-2 crisis. We could not find significant effects for the relationship between mother’s perceived everyday stress and the children's behavioral problems.

**Conclusions:**

Analyses illustrate the effects on children's mental distress during everyday life in the SARS-CoV-2 pandemic. Future research needs to identify influencing factors with regard to political, economic and social restrictions, in order to prevent children’s mental health problems.

## Introduction

The current situation caused by the SARS-CoV-2-pandemic is associated with serious losses for everyone and has been affecting social life, politics, the economy and the media worldwide (economic shutdown, contact restrictions, restriction of public life) [1].

The associated effects and consequences of the SARS-CoV-2-pandemic pose a major challenge, especially for children and their parents. In order to combat the SARS-CoV-2-pandemic, preventive isolation and social distancing strategies were developed and implemented to contain the risk of a wave of infection around the world, which started in mid-March 2020 and are still ongoing (such as regional and national containment measures or lockdowns) [2]. This means that families face a number of challenges (sudden closure of schools and childcare facilities, the loss of community programs and jobs, increasing pressure from recession/unemployment, home schooling, lack of social support from grandparents for example) [3, 4].

Such epidemiologically required restrictions seem to be particularly stressful for families. Brooks and colleagues [5] point out that they bear a plethora of psychological burden, varied neuropsychiatric manifestations and psychosocial stigma [5]. An earlier study by Sprang and colleagues [6] showed that post-traumatic stress significantly rises in children after experiencing quarantine. Similarly, the probability of developing acute stress disorder and adjustment disorder increases [6]. In addition, Shen and colleagues [2] refer to the long-term adverse consequences for children and adolescents [2], whereby the type and extent of pandemic-associated effects depend on many factors, such as developmental age, special needs, the mental health status and even the economic situation [3, 7–9].

Initial studies showed that the quality of life of children and adolescents in Germany decreased during the pandemic because of changes and restrictions related to social life [10]. Being isolated, fearing for one's grandparents and lacking contact with friends causes an immediate and persistent psychosocial effect for children due to the drastic changes in their lifestyle, physical activity level and mental excursions [8, 9]. Thus, the current epidemiological restrictions have a verifiable influence on the emotional and social development of children and adolescents. Particularly younger children (3–6 years) seem to be affected, which can be seen in reference to studies providing evidence for stress symptoms, such as excessive clinging to one parent and increased anxiety. Older children (6–18 years) show behaviors such as increased inattention, anxiety and a persistent interest in the current situation regarding the SARS-CoV-2 pandemic [11]. Viner and colleagues [12] confirmed these findings and referred to increased irritability, inattentiveness and increased nervousness, as well as more intense contact with the caregiver, regardless of a child’s age [12]. Parents indicated to perceive their children as more insecure, anxious and isolated, when compared to prior to the current situation. They also report a surge in sleep disorders, nightmares, loss of appetite, restlessness, inattentiveness and fear of separation [13–16].

Finally, latest research shows health risks and fears associated with SARS-CoV-2 affect parental stress and, as a result, children’s well-being [17]. In addition, experience from previous economic recessions has shown, that factors like unemployment, decreasing income, excessive debt and parental history of psychological stress, pose a serious threat to the mental health of a family in terms of decreasing mental well-being, increasing rates of various mental disorders, parental substance-related disorders or suicidal behavior for example [3, 18–20]. Even in less stressful situations, interactions between a child and their parent impact a child’s development, for instance, through the development of internalizing [21] or externalizing problems [22] as well as emotional adjustment [23]. In particular, factors like parental mental health [22], marital distress [23], parenting behavior [24], and parent–child relationships [21] are influential.

The current pandemic evidently harbors the risk of increased psychological stress for parents. This is reinforced by factors of economic hardship, such as a reduction in the scope of parental employment and thus, difficulties in covering basic needs [16, 25]. However, especially in times of paramount stress and uncertainty triggered by a pandemic, young children in particular, urgently need a secure and stable family environment [26]. This study is substantial in examining how parental stress, even during minor stressful situations, affects children’s mental health during a stress-inducing time like the current pandemic. Therefore, this study offers a different perspective on the longitudinal influence of parental stress, indicating a significantly increased stress vulnerability for both parents and children.

Shen and colleagues [2] point out that this pandemic may continue to have long-term adverse consequences for children and adolescents compared to adults [11]. The well-being of children does not only depend on nutrition and medical care, but also on parental guidance [27]. One of the first studies to investigate psychological stress on account of the SARS-CoV-2 pandemic, provides empirical evidence for a positive relationship between the psychological stress of parents and that of their children [18, 25, 28]. Considering current research, we do not only take into account the psychological stress caused by the current pandemic, but equally refer to pre-pandemical perceived stress.

Therefore, our study aims to analyze the effects of maternal stress level since childbirth on children’s mental health during the SARS-CoV-2 pandemic. We hypothesize that children of mothers that have reported higher levels of daily stress since childbirth, are also at greater risk for mental distress.

## Methods

### Study design

TRANS-GEN is an interdisciplinary study consortium investigating the pathways leading to resilience or vulnerability in the transgenerational transmission of childhood maltreatment (CM) by focusing on psychological, biological and social factors, adopting a prospective approach. The study was funded by the Federal Ministry of Education and Research and was approved by the Ethics Committee of Ulm University.

After recruiting within the maternity unit of the Ulm University Hospital, all mother–child dyads were followed up with thrice: 3 months (t1), 12 months (t2) after birth and at age 3.

In order to measure the impact of the SARS-CoV-2-pandemic on a child's mental health, all participating mothers were asked to take part in a conducted online “SARS-CoV-2-pandemic survey” which was available from May 18–July 31 2020.

### Participants

Since October 2013, 533 mother–child-dyads have been recruited in the women’s hospital of the University Hospital of Ulm within 1–6 days after childbirth, later voluntarily participating in the screening interview. The following inclusion criteria were used for sample selection: age > 18, more than 37 weeks of pregnancy, sufficient knowledge of the German language, no complications during childbirth or health problems of mother and/or child, as well as no current drug use or a history of severe medically diagnosed lifetime psychotic disorders or current infections. All mothers were questioned about the relevant criteria before the start of the study. 240 mother–child-dyads met the criteria and could be invited for a follow-up appointment 3 months (t1) postpartum in both a laboratory and a home visit. 158 mother–child-dyads participated in an additional laboratory and home visit when the child was around 12 months old (t2) and in a further home visit, where the child was approximately 3 years of age. The reasons for drop-outs of mother–child-dyads between t0 and t3 varied and ranged from personal reasons and a lack of interest, to missing time windows for further participation in the study. For 158 mother–child-dyads we acquired a full dataset, with measurements from t0 to t3. Accordingly, all dyads with a complete dataset were asked to fill in the online “SARS-CoV-2-pandemic survey”. 73 mothers completed the online survey until the end of July 2020, consequently eligible to partake in the current study.

Mothers' age at the time of the “SARS-CoV-2-pandemic survey” was between 31 and 46 years [mean 38.20 years (SD 4.06 years)] and children’s ages ranged from 4.98 to 7.14 years [mean 6.03 years (SD 0.61 years)]. 47.83% of participating children are female. 79.1% of the mothers reported to be married or living in a partnership, and 89.6% of all mothers had German citizenship. Mothers' level of education in comparison to the educational background of the German population showed, that 72.8% had a grammar school degree (13 years of school), 20.7% a secondary school degree (10 years of school), 5.4% a basic secondary school degree (9 years of school) and 1.1% no school diploma (see Table 1). Unfortunately, no data on which participating families have suffered from Covid-19 is available to us.

**Table 1:** Descriptive statistics

Variable	Mean	S. D	Min	Max	%
Emotional problems of child during SARS-CoV-2 pandemic	6.93	2.12	5	13	
Emotional problems of child before SARS-CoV-2 pandemic	6.17	1.46	5	10	
Behavioral problems of child during SARS-CoV-2 pandemic	3.14	1.07	2	6	
Behavioral problems of child before SARS-CoV-2 pandemic	1.48	.60	2	3	
Hyperactivity of child during SARS-CoV-2 pandemic	2.97	1.31	2	6	
Hyperactivity of child before SARS-CoV-2 pandemic	2.63	.94	2	6	
Maternal perceived everyday stress	61.96	24.79	20	145	
Age of mother during the survey	38.2	4.06	31	46	
Adverse childhood experiences of mother	6.62	2.90	5	17	
Change in working hours	− 0.06	0.65	− 1	1	
Decrease in income	0.06	0.23	0	1	
Social support	7.80	2.21	0	10	
Age of child during the survey	6.03	.61	4.98	7.14	
Marital status: married					79.1
German citizenship					89.6
Level of education of mother					
Grammar school degree					72.8
Secondary school degree					20.7
Basic secondary school degree					5.4
No school diploma					1.1

### Measures

The online “SARS-CoV-2-pandemic survey” included socio-demographic questions, such as the age of the mother, their educational level, occupation, marital status, number of persons under the age of 18 living in the household and the number of her own children. In addition, it was recorded whether the mother and her potential partner were currently working in a systemically relevant area (professional groups, which contribute to maintaining the economy, health system or basic services) and whether the household income had decreased by more than a quarter since the beginning of the SARS-CoV-2 pandemic [29, 30].

By using the German short version of the Childhood Trauma Questionnaire [31, 32], all mothers were screened for maternal childhood maltreatment. The retrospective self-report questionnaire was conducted at the first measurement point (t0), assessing experiences of physical, sexual and emotional abuse, as well as physical and emotional neglect before the age of 18 [33]. With five items for each subscale measured by a 5-point Likert scale, the subscale scores subsequently range from 5 to 25, the CTQ assesses the load of maltreatment from “none” maltreatment experiences (25 points) over “minimal” to “extreme” [34].

Children’s mental health was assessed via selected items of the German version of the Strengths and Difficulties Questionnaire (SDQ) [35], an established short behavioral screening questionnaire completed by the parents. The SDQ addresses positive and negative behavioral attributes of children on five scales regarding both strengths and difficulties. The questionnaire is made up out of 25 items, each of its five scales (emotional problems, externalizing behavioral problems, hyperactivity/attention problems, problems with peers and prosocial behavior) contains five items, which are rated on a 3-point Likert scale. For the “SARS-CoV-2-pandemic survey” we selected items concerning emotional problems, externalizing behavioral problems and hyperactivity/attention problems. The item-values were added up individually for each dimension to achieve operationalization. Following items were selected for the emotional problems scale: “Frequently complains of headache, stomach ache or nausea”, “Has a lot of worries, often appears depressed”, “Often unhappy or depressed; often cries”, “Nervous or clinging in new situations; easily loses self-confidence” and “Has many fears; is easily afraid”. For the scale externalizing behavioral problems the item “Has tantrums often; is short-tempered” and “Generally obedient, doing mostly what adults ask” were selected as corresponding items. “Restless, overactive, can’t sit still for a long time” and “Constantly fidgety” were chosen as items for the hyperactivity/attention problems scale. We decided not to measure the scale “problems with peers” and “prosocial behavior” due to the pandemic-associated restrictions in the context of school and kindergarten closures, as well as the limitation of social contacts outside the family.

The maternal perceived everyday stress was measured starting with the birth of the child at t1, further measures were taken at t2 and t3 using the Perceived Stress Scale 14 [36]. The PSS14, the original 14 item-version, is a widely and well-established self-report scale measuring perceived stress on a 5-point response scale, consisting of seven negative and seven positive items. High values indicate a high level of perceived stress. Since the PSS is not a diagnostic tool, no limit values exist. The level of perceived everyday stress was recorded for all participating mothers during the first (t1), second (t2) and third follow-up (t3). In order to provide an overall measure of the maternal perceived everyday stress, we added up z-standardized values from all three measurement times. The stress measure therefore forms the longitudinally recorded daily stress level of the mother, beginning after giving birth and continuing on to a child's third year of life. Hence, it must be clearly differentiated from the pandemic-related stress level of the mother.

In our model we control for the age of the mothers, changes in working hours and income as well as social support. Changes in working hours and income was assessed by the questions “Has the income available in your household fallen by more than a quarter since the beginning of the crisis?” and “Has your amount of work changed since the beginning of the crisis?” Changes in working hours were inquired utilizing the following answering options: “Yes, I work more”; “Yes, I work less”; “No, I work the same amount”. Changes in income were gathered with a binary coded answer. The scope of support that mothers experienced was measured using the following statement “I have people who I can talk to about my problems and who understand me” on a ten-point-scale.

## Model and estimation

Tables 1, 2 show the descriptive statistics for the (standardized) variables as well as the correlations. Table 1 displays values for emotional, behavioral and hyperactivity problems of a child both before and during the pandemic. For all three dimensions we find higher mean values at the latter observation time. We standardize variables X_Std_ according to Formula 1 below with the mean µ and standard deviation σ.

$$Formula\,1:X_{{Std}} = \frac{{X - \mu }}{\sigma }$$

**Table 2:** Descriptive statistics of standardized variables and intercorrelation

Variable	Mean	S. D	Min.	Max.	4	5	6	7	8	9
Emotional problems of child during SARS-CoV-2 pandemic	− 3.87*10^−8^	3.36	− 3.10	9.76						
Behavioral problems of child during SARS-CoV-2 pandemic	− 4.60*10^−8^	1.69	− 1.75	4.90						
Hyperactivity of child during SARS-CoV-2 pandemic	3.64*10^−8^	1.91	− 1.40	4.43						
Maternal perceived everyday stress	0.16	2.58	− 4.19	9.05	1.00					
Age of mother	− 7.62*10^−9^	1	− 1.77	1.92	0.18	1.00				
Adverse childhood experiences of mother	6.95*10^−9^	1	− .56	3.58	0.44***	0.02	1.00			
Change in working hours	7.86*10^−9^	1	− 1.46	1.63	0.12	− 0.08	0.02	1.00		
Decrease in income	− 1.16*10^−8^	1	− 0.24	4.10	0.01	− 0.11	− 0.12	− .017	1.00	
Social support	9.19*10^−9^	1	− 3.52	.99	− 0.22^#^	.16	− 0.30**	− 0.001	− 0.22*	1.00

^#^p < 0.1; *p < 0.05; **p < 0.01; ***p < 0.001

For our estimation we applied Tobit Models because of our limited dependent variables [37]. The use of Tobit Models refers “*to regression models in which the range of the dependent variable is constrained in some way*” [42; p. 3]. The following six equations illustrate the models we worked with for the empirical estimation. Equations (1)–(6) correspond to the models 1–6 in Tables 3, 4. Abbreviations correspond to the list below:

1 $${\text{EPC}} = \alpha + \beta _{1} \,{\text{MPS}} + \varepsilon .$$

2 $${\text{EPC}} = \alpha + \beta _{1} \,{\text{MPS}} + \beta _{2} \,{\text{AM + }}\beta _{3} \,{\text{ACE + }}\beta _{4} \,{\text{CW + }}\beta _{5} \,{\text{DI + }}\beta _{6} \,{\text{SSU}} + \varepsilon .$$

3 $${\text{BPC = }}\alpha {\text{ + }}\beta _{1} \,{\text{MPS + }}\varepsilon {\text{.}}$$

4 $$BPC = \alpha + \beta _{1} \,{\text{MPS + }}\beta _{2} \,{\text{AM + }}\beta _{3} \,{\text{ACE + }}\beta _{4} \,{\text{CW + }}\beta _{5} \,\,{\text{DI + }}\beta _{6} \,{\text{SSU + }}\varepsilon {\text{.}}$$

5 $${\text{HCC = }}\alpha + \beta _{1} \,{\text{MPS + }}\varepsilon {\text{.}}$$

6 $${\text{HCC = }}\alpha + \beta _{1} \,{\text{MPS + }}\beta _{2} \,{\text{AM + }}\beta _{3} \,{\text{ACE + }}\beta _{4} \,{\text{CW + }}\beta _{5} \,{\text{DI + }}\beta _{6} \,{\text{SSU + }}\varepsilon {\text{.}}$$

with, *ACE* Adverse childhood experiences of mother, *AM* Age of mother, *BPC* Behavioral problems of child during SARS-CoV-2-pandemic, *CW* Change in working hours, *DI* Decrease in income, *EPC* Emotional problems of child during SARS-CoV-2-pandemic, *HCC* Hyperactivity of child during SARS-CoV-2-pandemic, *MPS* Maternal perceived everyday stress, *SSU* Social support.

**Table 3:** Results for Tobit estimations (model 1–4)

Dependent variable	EPC	EPC	BPC	BPC
Independent variable	Model 1	Model 2	Model 3	Model 4
Maternal perceived everyday stress	0.33* (0.15)	0.43* (0.18)	0.14^#^ (0.08)	0.14 (0.10)
Age of mother		− 0.37 (0.45)		0.13 (0.24)
Adverse childhood experiences of mother		− 0.16 (0.50)		− 0.24 (0.26)
Change in working hours		0.02 (0.71)		− 0.64^#^ (0.38)
Decrease in income		4.09 (2.44)		1.25 (1.29)
Social support		0.03 (0.50)		− 0.24 (0.27)
Constant	0.35 (0.39)	0.33 (0.43)		− 0.02 (0.23)
N	73	58	73	58
VIF		1.18		1.18
Pseudo R^2^	0.01	0.03	0.01	0.03
LR Chi^2^ test	4.41	9.23	3.16	7.93

^#^p < 0.1; *p < 0.05; **p < 0.01; ***p < 0.001

**Table 4:** Results for Tobit estimations (model 5–6)

Dependent variable	HCC	HCC
Independent variable	Model 5	Model 6
Maternal perceived everyday stress	0.36*** (.09)	0.34** (0.10)
Age of mother		− 0.65* (0.25)
Adverse childhood experiences of mother		0.10 (0.28)
Change in working hours		0.10 (0.39)
Decrease in income		3.04* (1.41)
Social support		0.39 (0.28)
Constant	0.17 (0.23)	0.13 (0.24)
N	73	58
VIF		1.18
Pseudo R^2^	0.05	0.09
LR Chi^2^ test	14.39	21.54

^#^p < 0.1; *p < 0.05; **p < 0.01; ***p < 0.001

## Results

Table 2 shows values for correlations between independent and control variables below a level of 0.5. To test for multicollinearity we calculated the variance inflation factor (VIF) for our models (VIF = 1.18). With reference to literature [38] that suggested a VIF below 10 to prevent multicollinearity. Tables 3, 4 show the results for the Tobit Models. Due to missing values sample sizes in our models vary. We found a positive and significant effect of the maternal perceived everyday stress on children’s emotional problems during the pandemic both in the full-model (Model 2 with β_1_ = 0.43; p < 0.05) and in Model 1 (β_1_ = 0.33; p < 0.05).

Model 3 and 4 display the results for the relationship between the mother’s perceived everyday stress before the pandemic and the children’s behavioral problems during the SARS-CoV-2 crisis. Based on the results in Table 3 we could only find an effect for Model 3 (β_1_ = 0.14; p < 0.1). Model 5 and 6 illustrate the results for the influence of the mother's perceived stress before the pandemic on the children’s hyperactivity levels during the SARS-CoV-2 crisis. The assumption of a positive relationship could be supported for both Model 5 (β_1_ = 0.36; p < 0.001) and Model 6 (β_1_ = 0.34; p < 0.01). A decrease in income showed a positive and significant effect on the hyperactivity levels of a child (β_5_ = 3.04; p < 0.05). Additionally Model 6 provided support for a negative and significant relationship between the mother’s age and the children’s hyperactivity (β_2_ = − 0.65; p < 0.05).

## Discussion

The current SARS-CoV-2-pandemic affects children adolescents and their families in exceptional ways. The restrictions in everyday life required to contain the pandemic such as school closures or home schooling (UNESCO) restricted private shielding or quarantine but also the cancellation of out-of-home leisure time activities [8,9,39] can overstain already charged family situations easily [3]. Furthermore external support offered by other family members as well as institutional social systems have fallen away [3].

However a stable and secure family environment to which mentally healthy parents essentially contribute is a strong protective factor for children [26] in their everyday life. Current research focusing on pandemic-related effects on young children between 3 and 6 years of age shows that in contrast to older children they are significantly more likely to experience stress symptoms in their emotional and social development [12]. The findings of our study contribute to existing literature by considering a lack of pre-pandemic protective factors and investigating its influence on mental health during the pandemic. Current studies on mental stress in parents and their children mainly target the stress level of parents and their children caused by the pandemic with regard to a possible link [40]. Existing studies differ from the present study discussed here because the focus is on longitudinally recorded daily maternal perceived stress. Since it was measured across the children’s first years of life starting at their birth the maternal perceived stress is not solely limited to causes of stress due to the pandemic. To the best of our knowledge the present study is the first one estimating the relationship between longitudinally assessed maternal mental stress before the SARS-CoV-2 crisis and children’s mental health during the pandemic.

In our study we illustrate that children of mothers with a higher pre-pandemic level of stress show significantly higher levels of mental distress during the pandemic. In detail we find empirical evidence that children of stressed mothers have significantly higher values regarding their emotional stress level as well as increasing hyperactivity and attention problems during times of the pandemic. Looking at the aggregated behavioral problems of children during the SARS-CoV-2 pandemic reveals that the extent of maternal perceived stress starting at their child’s birth has a positive effect. This highlights that the mothers’ individual stress experience through life is perceived as unpredictable uncontrollable and overloading. Regardless of the current crisis caused by SARS-CoV-2 it directly influences a child’s stress experience and behavioral attributes. Therefore it is of utmost importance that the current parental stress is viewed as a risk factor in relation to positive coping skills in regard to children’s stress caused by the pandemic [40]. Our results are consistent with the findings of Davico et al. [28] Patrick et al. [25] and Crescentini et al. [41] who detected a positive relationship between the experienced psychological stress of parents and that of their children [25, 28, 41]. However in contrast to the formerly mentioned studies we contribute to literature by estimating models based on longitudinal data which captures the maternal stress level examined from childbirth on. Thus we are able to map the course of maternal stress development over time. In summary findings show that the mother’s psychological stress exposure regardless of its temporal origin has a considerable influence on a child’s experience of stress during the pandemic which highlights the relevance of a secure and stable foundation akin to a child's caregiver.

There is a lack of literature estimating the influence of a combined measure of both the effects of pre-pandemic stressors and pandemic-related distress on health outcomes. Since an interaction of these effects can be assumed future research should target this issue. Further results could provide a basis for long term practical measures.

Nevertheless we have to address several basic limitations of our study. Firstly our sample is restricted to participants from an online survey which is why the findings cannot be considered representative for the general public and therefore not generalizable for all families. This limitation is also related to the inherent characteristics of our sample as a larger number of participants were highly educated which has to be considered when interpreting the data or comparing it to other studies. Moreover we focus on a small sample due to the short time period of data collection at the beginning of the pandemic. Further research could estimate models with larger sample sizes to verify results. Lastly the assessment of both maternal perceived everyday stress and behavioral attributes of children were retrospectively self-reported by mothers. Thus answers may be biased by social desirability. Furthermore the participating mothers were asked about their own mental health as well as that of their children. The psychological stress experienced by mothers can impact how they perceive the mental health of their children. With respect to this the external validity of one of these two candidates should be considered in further research. In addition only the maternal daily perceived stress in the period before the SARS-CoV-2-pandemic was recorded in a longitudinal manner. Therefore it would be useful to conduct another longitudinal measurement reflecting the mothers’ stress levels during the current pandemic. Finally the fact that the assessment of positive and negative behavioral attributes of children is solely based on individual items of the SDQ is also a limitation of this study. The currently discussed findings must therefore be interpreted with caution when compared to other existing literature.

## Conclusion

Preventive isolation and social distancing strategies cause families to encounter a large number of different challenges. Especially in times of crisis children once again need a secure and stable family environment. Maternal perceived everyday stress is a significant predictor for increasing a child’s mental distress during the SARS-CoV-2-pandemic. These findings highlight the importance for future research to identify risk and protective factors to prevent adverse mental health outcomes for children in an exceptionally stressful situation like the current pandemic.

## Data Availability

The datasets analysed during the current study are available on a database of the University Hospital of (Ulm).

## Abbreviations

ACE: Adverse childhood experiences of mother
AM: Age of mother
BPC: Behavioral problems of child during SARS-CoV-2-pandemic
CW: Change in working hours
DI: Decrease in income
EPC: Emotional problems of child during SARS-CoV-2-pandemic
HCC: Hyperactivity of child during SARS-CoV-2-pandemic
MDC: Mental distress of child during SARS-CoV-2-pandemic
MPS: Maternal perceived everyday stress
SSU: Social support
